# RAID: Retrieval Augmented Intrusion Detection for Cyber Physical Systems

**DOI:** 10.3390/s26216727

**Published:** 2026-10-24

**Authors:** Alberto Moccardi, Rajib Chandra Ghosh, Marcello Pelosi, Flora Amato

**Affiliations:** Department of Electrical Engineering and Information Technology (DIETI), University of Naples Federico II, Via Claudio 21, 80125 Naples, Italy

**Keywords:** intrusion detection, resilient artificial intelligence, retrieval-augmented generation

## Abstract

This paper presents a Retrieval Augmented Intrusion Detection system (RAID), a three-stage framework for interpretable AI-driven intrusion detection in Industrial Control Systems (ICS). Existing ICS detectors flag anomalies without explaining them, and LLM-based explanations are rarely verified against the detector’s rules or linked to past incidents. Specifically, a resilient isolation forest algorithm is used for its ability to generate structured anomaly interpretations and to mitigate the effects of dangerous data drift. A grounded physics-aware feature engineering process extends the baseline capabilities of this algorithm, including counterfactual evaluation, contrastive analysis, causal chain identification, and redundancy inspection of data streams. Subsequently, a fleet of Large Language Models (LLMs) is used to generate deep natural-language explanations for each anomaly log, under the guidance of an independent LLM orchestrator that produces a final incident report. Finally, a Retrieval-Augmented Generation framework embeds the historical patterns of the recovered events into an accessible index that contains all the log interpretations over time. The entire framework is validated at each stage on the HAI-21.03 dataset: among nine unsupervised detectors, the Isolation Forest provides the most balanced performance (F1 = 0.409, MCC = 0.393, eTaPR F1 = 0.773); 87% of the propositions generated by the language agents exactly match the underlying detection rules; and the retrieval stage reaches 0.998 faithfulness and 0.782 answer correctness.

## 1. Introduction

Industry 4.0 represents a global technological and social transformation in which information is incorporated directly into components [1], based on the integration of physical and digital systems and enabled by technologies such as IoT, Big Data, and Artificial Intelligence. Real-time analysis of data generated during operation allows for the optimization of production processes, enabling autonomous, flexible, and adaptive systems. The fundamental technological core of Industry 4.0 consists of Cyber-Physical Systems (CPS). Specifically, a CPS allows a physical system to be equipped with a virtual system, enabling the analysis of data collected from the physical world. In this way, it is possible to make decisions that influence the physical world again. The physical system consists of physical processes, sensors, and actuators. The cyber system includes computing and control centers and communication networks. The computing and control center is responsible for receiving data measured by the sensors and analyzing it in order to make decisions that ensure the correct execution of physical processes. The communication network, on the other hand, acts as an intermediary between the physical world and the computing and control center, conveying information from the sensors to the latter and then transmitting decisions to the actuators [2]. Favored for the operational advantages they offer, the rapid spread of CPS is nevertheless accompanied by significant security challenges. Unlike traditional IT attacks, the ultimate goal in CPS is often kinetic compromission. In these cases, the goal is to induce actuators to act outside the operational limits defined by the control system, causing permanent physical damage or disabling safety logic. A widely documented example in the literature is packet replay attacks. In this type of attack, a legitimate network command is simply intercepted and then re-injected into the network at a time when that action is harmful, exploiting the absence of intrinsic authentication and time validation mechanisms in many legacy industrial protocols. Misaligning the control logic from the physical state of the plant in this way can cause serious physical and mechanical damage without the system detecting any anomaly in the communication format. Additional attack vectors include firmware reverse engineering techniques and dynamic analysis of software processes, which allow attackers to decode proprietary protocols and understand the exact correspondence between computer instructions and the mechanical reactions of the plant. This type of knowledge can then be reused through packet crafting to create malicious payloads. Following the proliferation of accessible tools that operate in this way, it is possible to note that, while the sophistication of attacks is increasing, the technical knowledge required of attackers to carry out such attacks has often decreased, giving rise to a scenario of systemic risk.

With the proliferation of digital infrastructure, network technologies, and data generation, traditional rule-based intrusion detection systems are not sufficient to counter the rising cyber threats. Signature-based intrusion detection systems are not flexible in detecting zero-day exploits and polymorphic malware [3]. This has led to the adoption of machine learning and AI technologies in intrusion detection systems to improve their adaptability in detecting anomalous behavior [4].

Anomaly detection algorithms in AI-based intrusion detection systems (IDS) range from simple statistical methods to very complex deep learning methods [5]. Previous methods include statistical profiling and threshold-based methods [6], followed by distance-based and clustering methods such as k-NN and k-means clustering algorithms [7]. Density-based methods such as DBSCAN further enhance unsupervised anomaly detection methods [8]. Supervised methods such as SVM and Random Forests enhance the accuracy of the detection algorithms using labeled data [9]. More complex methods such as Autoencoders, Recurrent Neural Networks (RNN), and LSTM networks are also employed to detect intrusion in the network [10,11], including LSTM models with metaheuristic optimization [12]. Transformer-based detectors then replaced recurrent models in many IDS settings, since self-attention captures long-range temporal and inter-variable dependencies [13,14,15]. Scaled-up transformers led to the Large Language Models discussed below. Although the accuracy of the intrusion detection algorithms has been enhanced using AI-based methods, it has also created a high level of complexity in understanding the algorithms, thereby increasing the gap between the algorithms and human understanding.

This interpretability gap is a key challenge in human-in-the-loop environments for intrusion detection systems. Machine learning models often produce high-dimensional embeddings, probability-based anomaly scores, or feature importance vectors that are not interpretable for human analysts. Explainable AI research aims to reduce this problem by increasing transparency and interpretability in machine learning models [16,17]. However, even after using XAI methods, converting these outputs into a coherent narrative for forensic analysis is a big challenge in many cybersecurity scenarios.

Language Models (LMs) appear to offer an attractive solution in bridging this identified gap. State-of-the-art LMs are based on the transformer architecture introduced in “Attention Is All You Need” by Ashish Vaswani et al. in [18], allowing for contextual learning using self-attention mechanisms. Large Language Models (LLMs) like BERT from Google [19] and GPT from OpenAI [20] are scaled versions of the transformer architecture, allowing for billions of parameters to be included in the model, thus offering improved reasoning, summarization, and contextual composition abilities. These models can interpret structured and unstructured data, thus making them an attractive choice as an intermediary semantic layer between diagnostic systems and human users.

As part of an artificial intelligence-driven intrusion detection system (IDS) stack, large language models (LLMs) can be utilized as an interpretive tool for complex rule-based “IF” diagnostics and machine learning-based anomaly detection. LLMs can be used not as a replacement but as a tool to provide contextualization, correlation, and explanation of IDS-generated alarms, thus improving usability and human understandability [21,22,23].

On the other hand, “Retrieval-Augmented Generation” (RAG), as defined in a recent paper by Patrick Lewis et al. [24], enhances the reliability of large language models with the aid of external knowledge retrieval systems [25]. In the context of intrusion detection systems, a system based on the RAG model will be capable of retrieving relevant threat intelligence, historical logs, vulnerabilities, or compliance data before generating its interpretive results. This makes it a complete IDS forensic assistant.

Despite these benefits, there are also some challenges associated with the integration of large language models (LLMs). The major challenges are those of hallucination and information dilution [26,27]. In this regard, it is important to understand that hallucination refers to the creation of information that looks correct but is actually incorrect. Such an issue would be risky for forensic analysis. Information dilution refers to a situation where important technical information is simplified during natural language abstraction. Such information might be important for attack-related evidence. To mitigate these risks, it is important to include grounding methods, retrieval verification, and confidence scoring.

To the best of our knowledge, no existing framework connects an unsupervised ICS detector, LLM reports verified against the detector’s rules, and a searchable memory of past incidents.

The objective of this work is to propose a framework that covers the full chain from machine-learning-based detection, through natural-language interpretation, to the construction of a queryable historical knowledge base. The main contributions of this work are summarised as follows:

- A drift-resilient detection stage that combines physics-informed feature engineering, a regime-aware Isolation Forest, and a clean-window adaptive threshold, and that equips each detected event with a six-component structured explanation (Section 3.1, Section 3.2 and Section 3.3).
- A multi-agent LLM pipeline that translates the structured explanations into natural-language incident reports, in which an independent Judge Agent verifies each generated proposition against the formal detection rules, allowing the hallucination rate to be quantified (Section 3.4).
- A retrieval-augmented forensic memory that indexes the incident reports in a FAISS vector store and combines dense retrieval with maximal marginal relevance (MMR) re-ranking, so that operators can query previous incidents (Section 3.5).
- A stage-wise validation on the HAI-21.03 dataset, comprising a benchmark of nine unsupervised detectors under point-wise, event-level, and time-aware (eTaPR) metrics, a static versus drift-aware thresholding ablation, a judge-based assessment of explanation fidelity, and a RAGAS-based comparison of prompting strategies and retrieval baselines.

## 2. Dataset & Material

The dataset chosen as a reference for the experiment in this study is the HAI (HIL-based Augmented ICS) Security Dataset 21.03 [28]. The dataset was developed from a simulated realistic test environment consisting of four main processes (P). Boiler (P1) and Turbine (P2) were used together to simulate the operation of a steam-powered thermoelectric power plant. A water treatment process (P3) was used to simulate a pumped storage hydroelectric power plant. A fourth process (P4) consists of HIL (Hardware-In-the-Loop) simulation, which combines these three processes by harmonizing the signals so that they behave exactly as they would in a real industrial scenario. In order to operate the plant, a classic industrial automation cycle based on PLCs (Programmable Logic Controllers) has been set up. As is often the case, this is based on three essential elements: Process Variable (PV), i.e., the current reality read by the sensors; Set Point (SP), i.e., the target value, which can be set via a human–machine interface; Control Variable (CV), i.e., the command that the PLC sends to the “actuators” to ensure that the process variables align with the target expressed by the SP. The dataset presents both a normal (benign) scenario and a scenario under attack. In order to create the former, a program automatically and cyclically changed the reference set points, complying with security criteria and thus reflecting behavior similar to normal daily operations. To simulate the latter, however, 50 cyber attacks were intentionally carried out, and the system’s reactions following its tampering were then recorded. Specifically, 25 attacks were single attacks, while the remaining 25 were simultaneous combined attacks. The dataset used is structured in 84 columns. In addition to the timestamp column, there are 78 columns relating to data collected by the SCADA system. The final four columns, on the other hand, are reference labels. These indicate whether or not the system was under attack, with reference to the individual processes P1, P2, P3 and, in a generic manner, using a general variable. The P4 variable, on the other hand, lacks a dedicated label for anomalies. The three training files contain normal operation only and are used for fitting and threshold calibration, while test1 and test2 (25 attacks) are used for evaluation. To assess generalization, the detection stage is also evaluated on HAIEnd 23.05 [29], which records 225 internal signals of the boiler control system with 52 attacks. Its tags do not map onto setpoint/process-variable pairs, so only the generic residual features can be built on it.

## 3. Methodology

**Figure 1:**
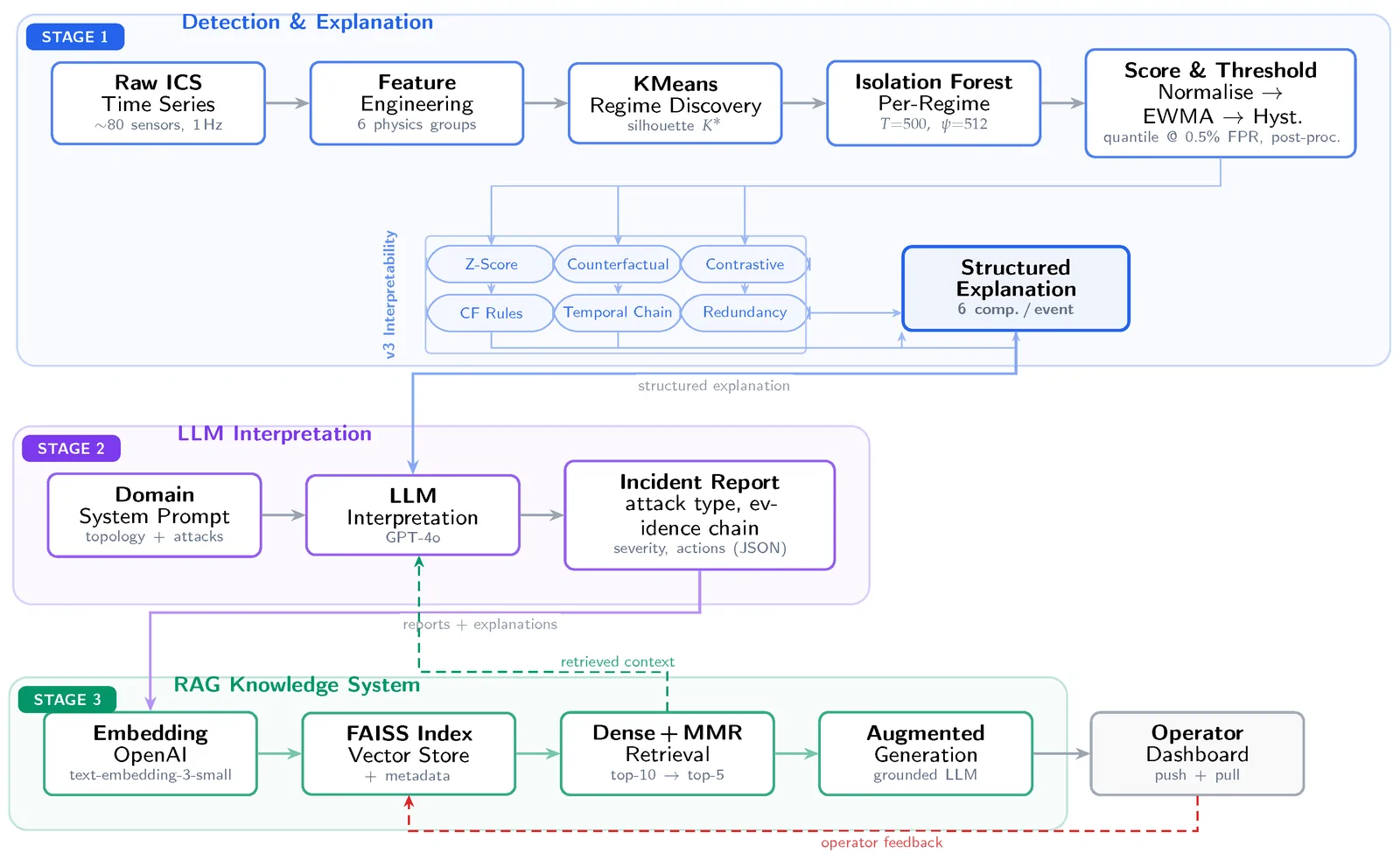
Three-stage methodology overview. **Stage 1** (blue): raw ICS time series undergo physics-informed feature engineering (six groups, ∼330 features), KMeans regime discovery, per-regime Isolation Forest scoring, EWMA-smoothed thresholding, and multidimensional explanation extraction (six complementary components per event). **Stage 2** (purple): a domain-prompted LLM transforms structured explanations into natural-language incident reports. **Stage 3** (green): a RAG system embeds and indexes all historical explanations, and dense retrieval with MMR re-ranking augments the LLM context for new incidents, while operator corrections feed back into the knowledge base.

The proposed methodology follows a three-stage workflow, as shown in Figure 1.

In Stage 1, raw Industrial Control System (ICS) streams are transformed into detected anomaly events, each with a corresponding structured multidimensional explanation through a resilient Isolation Forest (IF) algorithm.

In Stage 2, a Large Language Model is employed to convert these explanations into natural language incident reports, including domain knowledge from the ICS domain.

Finally, in Stage 3, a knowledge base is integrated via a Retrieval-Augmented Generation (RAG) that indexes all explanations and reports, thus allowing for querying of previous incidents to provide contextually aware diagnostic support to operators. The structure of this methodology section presents:

- An overview of the feature engineering pipeline (Section 3.1)
- An introduction to regime-aware isolation forest with multidimensional explanation extraction (Section 3.2)
- Resilient isolation forest (RIF) via Adaptive Thresholds (Section 3.3)
- Language model for complex logs interpretation (Section 3.4)
- RAG System for intrusion forensics (Section 3.5)

### 3.1. Physics-Informed Feature Engineering

Raw sensor readings carry limited discriminative power against stealthy attacks that maintain values within plausible ranges. We augment the raw signals with domain features, each encoding a different aspect of expected process behaviour.

We consider six feature engineering procedures, which correspond to the structure of the cyber-physical data: targets/measures, commands/responses, and inter-sensor consistency. Each procedure produces a different feature group, constructed from a small set of general-purpose operations on the time series: the difference, the smoothed version, the rolling statistics, the correlation, and the deviation from a slowly changing baseline. Hence, the feature engineering methodology is applicable to the analysis of other plants and their respective datasets, using the available signals or their subsets that play an analogous role to the signals used in this study.

*Control features* (Group 1): These features describe the state of the feedback control loops. For the setpoint $s_{k}$ and the respective process variable $p_{k}$, the tracking error is defined as

(1) $$e_{k}\left(t\right)=s_{k}\left(t\right)-p_{k}\left(t\right),$$

and augmented to four features: (i) the smoothed magnitude of the tracking error to highlight offsets; (ii) the change from the previous step to highlight sudden variations; (iii) the rolling variability of the tracking error to highlight instability; (iv) the standardised tracking error relative to its recent history.

*Rationale:* Spoofing of the sensor or tampering with the setpoint is likely to result in a tracking error that is larger, more sustained, and/or more variable than expected, violating the normal relationship between the setpoint and the measured variable.

*Actuator features* (Group 2): These features evaluate the behavior of the actuators in response to the controller demands $d_{j}$ and the feedback/position $z_{j}$ using the mismatch.

(2) $$m_{j}\left(t\right)=d_{j}\left(t\right)-z_{j}\left(t\right).$$

Following this, simple statistics on the values of $m_{j}$ are computed, such as magnitude, smoothness-based persistence, command change rate, and energy.

*Rationale:* The injection of commands, faulty actuators such as sticking actuators, and the manipulation of feedback increase the level of mismatch between commanded and actual systems and can cause this to persist.

*Thermodynamic features* (Group 3) check for consistency with fundamental physical laws by examining the residuals of relationships between slowly varying variables.

*Rationale:* Attacks tend to affect only a small fraction of the total signals. Physically coupled signals should tend to co-vary. The manifestation of the impact of manipulated signals should appear as significant residuals.

*Cross-process features* (Group 4) measure the level of coupling between systems by using lag difference-based descriptors for representative signals and correlation between setpoints and their measurements.

*Rationale:* Under normal conditions, there should be a stable level of coordination.

*State features* (Group 5) identify discrete operating states through indicators of actuator saturation, setpoint step flags, and a multi-jump index that summarizes simultaneous abrupt changes across multiple channels (approximately 12 features).

*Rationale:* Many attacks manifest as unexpected changes in modes, saturations, and/or jumps, which can often be identified much more readily through discrete indicators than through values.

*Generic residuals* (Group 6) provide broad coverage for all remaining channels using time-series transforms per sensor (e.g., lags, deviation from a smooth baseline, short-horizon momentum) (∼260 features).

*Rationale:* When detailed plant models or tag mappings are unavailable, generic temporal statistics still capture drifts, spikes, and rapid fluctuations that commonly accompany anomalies in diverse cyber–physical settings.

After the feature engineering process, the feature matrix contains approximately 330 columns. Table 1 summarises the groups.

***Regime discovery.*** The normal behavior of industrial processes is often multimodal and conditional on the process conditions. However, the global model would mix the normal modes, thereby decreasing detection performance. To identify the operational regimes, MiniBatch K-Means is employed exclusively on the standard dataset. In this study, *K* is chosen to maximise the silhouette score over K∈{2,…,20} on the randomly selected 50,000-point subsample, while the model is trained on the complete set of normal data. Only regime discovery uses these 20 raw signals, while each Isolation Forest is trained on the full feature matrix of Table 1.

### 3.2. Regime-Aware Isolation Forest

This study considers the Isolation Forest (IF) as the base anomaly detector. In detail, an ensemble of T=500 trees is trained, where each tree is learned from a random subsample of ψ=512 normal instances using random recursive partitioning.

The anomaly score for an instance *x* is defined as

(3) $$s\left(x\right)=2^{-E[h(x)]\;/\;c(\psi )}\;,\;c\left(\psi \right)=2H\left(\psi -1\right)-\frac{2(\psi -1)}{\psi }\;$$

and represent the expected path length of an unsuccessful search in a binary search tree (BST), with H(·) the harmonic number.

Separate IF algorithms are trained for each regime *r* (identified from the training data) using only normal instances after RobustScaler standardization and variance filtering ($\sigma^{2}>10^{-8}$).

***Preprocessing.*** Raw scores from different regimes occupy different scales. Percentile-rank normalization, calibrated on normal training scores, maps all scores to [0,3]:

(4) $$\tilde{s}\left(x\right)=\mathrm{clip}\;\left(\frac{s-q_{0.01}}{q_{0.99}-q_{0.01}},\;0,\;3\right).$$

Normalised scores are smoothed with an EWMA filter (α=0.10) applied identically on training and test data, without any label-based reset, ensuring consistent filtering semantics.

The detection threshold $\tau^{*}$ is the (1−ε) quantile of the smoothed normal training scores (80,000 random training samples, ε=0.5%), so no attack label is used.

Anomaly detection is refined by hysteresis (activate at $\tau^{*}$, deactivate at $0.9\tau^{*}$), gap merging (≤30 samples), and short-event removal (<5 samples).

Algorithm 1 summarises the pipeline.
**Algorithm 1** Regime-Aware IF Detection Pipeline**Input:** $X_{\mathrm{tr}},y_{\mathrm{tr}},X_{\mathrm{te}}$**Output:** ${\hat{y}}_{\mathrm{te}},{\bar{s}}_{\mathrm{te}}$  1:Select top-20 raw features by variance (regime clustering only)  2:KMeans($K^{*}$) on $X_{\mathrm{tr}}\left[y_{\mathrm{tr}}=0\right]$→ regime labels *r*  3:**for** each regime *r* with ≥200 normals **do**  4:    Fit Scaler $\phi_{r}$, variance mask $k_{r}$  5:    ${\mathrm{IF}}_{r}.\mathrm{fit}\left(\phi_{r}\left(X_{r}^{\mathrm{normal}}\right)\left[:,k_{r}\right]\right)$  6:    Store $\mu_{r},\sigma_{r},X_{r}^{\mathrm{normal}}$  7:**end for**  8:Fit global fallback ${\mathrm{IF}}_{g}$ on all normals  9:Score train per-regime, normalise, smooth10:$\tau^{*}\leftarrow$(1−ε) quantile of smoothed normal training scores11:Score test per-regime, normalise, smooth12:${\hat{y}}_{\mathrm{te}}\leftarrow$ Hysteresis → MergeGaps → RemoveShort

***Multi-dimensional explanation extraction.*** For each detected anomaly event, we extract six complementary explanation components that together constitute the structured input to the LLM stage.

*Z-score analysis.* At the peak-score sample, per-feature z-scores are computed relative to the regime-specific normal distribution:

(5) $$z_{j}=\frac{x_{j}-\mu_{r,j}}{\sigma_{r,j}}.$$

The top-8 features are reported with domain category, deviation direction, and severity (mild <2.5σ, moderate 2.5–4σ, significant 4–6σ, severe >6σ), alongside a temporal pattern label (spike, transient, sustained, prolonged) and trajectory (escalating, decaying, plateau).

*Counterfactual importance.* Each feature *j* is replaced by its regime mean $\mu_{r,j}$ and the score drop is recorded:

(6) $$\Delta_{j}=s\left(x\right)-s\;\left(x^{\left(j\leftarrow \mu_{r,j}\right)}\right),$$

directly answering *which features, if returned to normal, would most reduce the anomaly score*.

Pairwise synergy is quantified as

(7) $${\mathrm{synergy}}_{ij}=\Delta_{ij}^{\mathrm{joint}}-\left(\Delta_{i}+\Delta_{j}\right).$$

Positive values indicate complementary features; negative values indicate redundancy. From the top-5 features, a counterfactual rule is constructed:

(8) $$f_{1}{⋈}_{1}\mu_{r,1}\;\wedge \;\cdots \;\wedge \;f_{5}{⋈}_{5}\mu_{r,5},\;{⋈}_{j}=\left\{\begin{array}{cc} > & \mathrm{if}\;x_{j}>\mu_{r,j}\\ \leq & \mathrm{otherwise} \end{array}\right.$$

Negative deviations therefore produce upper-bound conditions.

*Contrastive explanation.* The k=5 nearest normal neighbours (KD-tree) yield an inverse-distance-weighted centroid $x^{*}$.

Features with the largest $|x_{j}-x_{j}^{*}|$ show how this anomaly differs from the closest normal operation.

*Temporal causal chain.* Per-feature z-scores across the event window (extended by 20 pre-event context samples) reveal the activation time $t_{j}^{*}$, defined as the first timestep where $|z_{j}|\;>\;2\sigma$. The propagation order is validated against a domain-specific causal graph encoding known physical cause–effect relationships. If an effect activates before its cause, a *causal violation* is flagged, which may indicate direct sensor manipulation rather than natural process disturbance. Since the labels do not identify the manipulated signal, this heuristic cannot be validated quantitatively and is presented to the analyst as a hypothesis.

*Redundancy grouping.* Features contributing overlapping anomaly signal are clustered greedily: starting from the highest-impact feature, those with pairwise redundancy exceeding a threshold are merged:

(9) $$1-\frac{\Delta_{ij}^{\mathrm{joint}}}{\Delta_{i}+\Delta_{j}}>0.5.$$

Each group is represented by its most impactful member and annotated with a collective score drop. Table 2 summarises the rationale for the six components.

### 3.3. Resilient Isolation Forest via Adaptive Thresholds

Although KMeans-based regime discovery is utilized for regime identification of the operating space, it is evident that such clustering of the space often does not account for the entire range of the ICS.

Minor changes in load and setpoint values, saturation of actuators, degradation of sensors, and even unmodeled dynamics will cause small changes in the distribution of the values of the anomaly score of the detector within a regime identified by KMeans clustering. This will cause a fixed threshold on the anomaly score of the detector to become increasingly miscalibrated as time progresses.

Therefore, we propose a different approach and directly utilize the decision boundary derived from the IF anomaly scores to construct a more flexible and effective detector.

***Core intuition.*** KMeans offers useful but limited operating condition discretization. It does not capture fine-grained variability. On the other hand, drifts in the IF scores immediately indicate operating condition variability. By deriving the threshold from recent score statistics, the detector becomes *self-adapting*, as the threshold tracks the score statistics while adapting to changing regimes.

Let ${\tilde{s}}_{t}\in R_{\geq 0}$ denote the raw IF score at time *t*. To stabilize high-frequency fluctuations while preserving consistent semantics between calibration and inference, we apply exponentially weighted moving average (EWMA) smoothing:

(10) $$s_{t}\;=\;\alpha \;{\tilde{s}}_{t}\;+\;\left(1-\alpha \right)\;s_{t-1},\;s_{0}={\tilde{s}}_{0},$$

where α∈[0,1] and $s_{t}$ is the score used for thresholding.

*Clean-window dynamic threshold.* As scores can change over time (e.g., due to operating mode changes), we require a time-varying IF threshold. However, using a naive dynamic threshold, where the threshold is derived from the statistics of *all* recent scores, can cause the threshold to *chase* attacks. Once abnormal scores are observed, the threshold increases, and the anomaly can be missed.

To avoid contamination, we maintain a rolling *clean buffer* $C_{t}$ that stores only scores currently judged *normal*. For a buffer size *W*, the update rule is

(11) $$C_{t}=\left\{\begin{array}{cc} \left(C_{t-1}\cup \left\{s_{t}\right\}\right){|}_{|\;\cdot \;|\leq W}, & s_{t}<\tau_{t},\\ C_{t-1}, & s_{t}\geq \tau_{t}, \end{array}\right.$$

where |·|≤W truncates the buffer to its most recent *W* elements. Once the buffer contains at least *M* clean samples, we estimate a local threshold from the clean-score distribution:

(12) $$\tau_{t}=\mu_{t}+k\;\sigma_{t},\;\mu_{t}=\mathrm{mean}\left(C_{t}\right),\;\;\sigma_{t}=\mathrm{std}\left(C_{t}\right),$$

where k>0 is a sensitivity parameter (higher values of *k* mean fewer alarms). When $|C_{t}|\;<\;M$, we instead employ a static calibrated threshold $\tau^{\mathrm{base}}$ based on normal scores under a target false positive rate. In our implementation, $\tau_{t}\leftarrow \left(1-b\right)\;\tau_{t}+b\;\tau^{\mathrm{base}}$, with k=3, W=300, M=50 and b=0.2.

*Event-consistent decisions.* Direct thresholding may lead to fragmented detections if the score is close to $\tau_{t}$ but varies over time. For this reason, we employ *hysteresis*, using different values for the alarm to be activated and to be deactivated. The alarm is activated if the score crosses the higher threshold $\tau_{t}$ but deactivated only if the score crosses the lower threshold $\rho \tau_{t}$ with ρ<1:

(13) $${\hat{y}}_{t}=\left\{\begin{array}{cc} 1, & a_{t-1}=0\;\wedge \;s_{t}\geq \tau_{t},\\ 0, & a_{t-1}=1\;\wedge \;s_{t}<\rho \;\tau_{t},\\ a_{t-1}, & \mathrm{otherwise}, \end{array}\right.\;a_{t}\equiv {\hat{y}}_{t},$$

where $a_{t}$ denotes the alarm state. Intuitively, the hysteresis prevents the alarm from flickering on and off too frequently, because the change in the score must be larger to turn the alarm off than to turn it on. Finally, we join separated detections with short intervals between them, removing short events below a minimum length.

### 3.4. Language Model for Complex Logs Interpretation

The goal of this phase is to transform the anomaly detection outputs, expressed in a mathematically formal manner, into explanations formulated in natural language, with the clear requirement of preserving quantitative rigor and causal validity. To manage complexity and ensure output robustness, the pipeline adopts an agent-based architecture. The primary level of the architecture consists of a fleet of eight independent agents operating in parallel, each dedicated to analyzing a specific anomaly metric provided as output from the previous step. These agents combine quantitative analysis skills with integrated RAG capabilities, effectively accessing a knowledge base containing sensor descriptions and their operating ranges. In doing so, each agent ensures that its local explanations are never purely mathematical or abstract, but are always physically grounded and anchored to operational reality. The eight specialists correspond to the eight fields into which the six components of Table 2 are serialised, since the counterfactual component yields both score drops (Equation (6)) and pairwise synergies (Equation (7)), and the temporal chain yields both activation times and the causal-violation check. Each specialist receives one field. The isolated reports generated by the specialists are not simply concatenated, but are instead transmitted to a higher-level Synthesizer Agent, whose task is to draft a cohesive, critical, and rigorously number-based overall discourse. The validation of the entire cognitive chain is entrusted to an independent agent, who uses Structured Outputs to impose a strictly constrained response format (JSON). Specifically, its task is to parse the global summary generated by the Synthesizer Agent, break it down into minimal logical propositions, and compare each of them with the original mathematical rules (Ground Truth). For each proposition, the Judge Agent deliberates:

- Objective accuracy score: (1 for exact match, 2 for misinterpretation, 3 if the statement cannot be associated with the rules).
- Logical justification (Rationale): A compelling explanation that motivates the score assigned, thus allowing the hallucination rate of the architecture to be quantified.

### 3.5. RAG System for Intrusion Forensics

In the preceding section, we introduced how we generate a short diagnostic interpretation condition from LLM anomaly logs for each operating cycle. However, for technicians and especially non-technicians, it is often very difficult to understand the overall operating behaviour and root causes from raw or isolated anomaly interpretation. To facilitate understanding of the operating behavior and root causes, we have introduced a specialized Retrieval-Augmented Generation (RAG) framework. When this framework is selected, their ability to access and precisely manipulate knowledge is more extensive than with other techniques, such as fine-tuning or pre-training the model, which store factual knowledge in their parameters [24].

**Figure 2:**
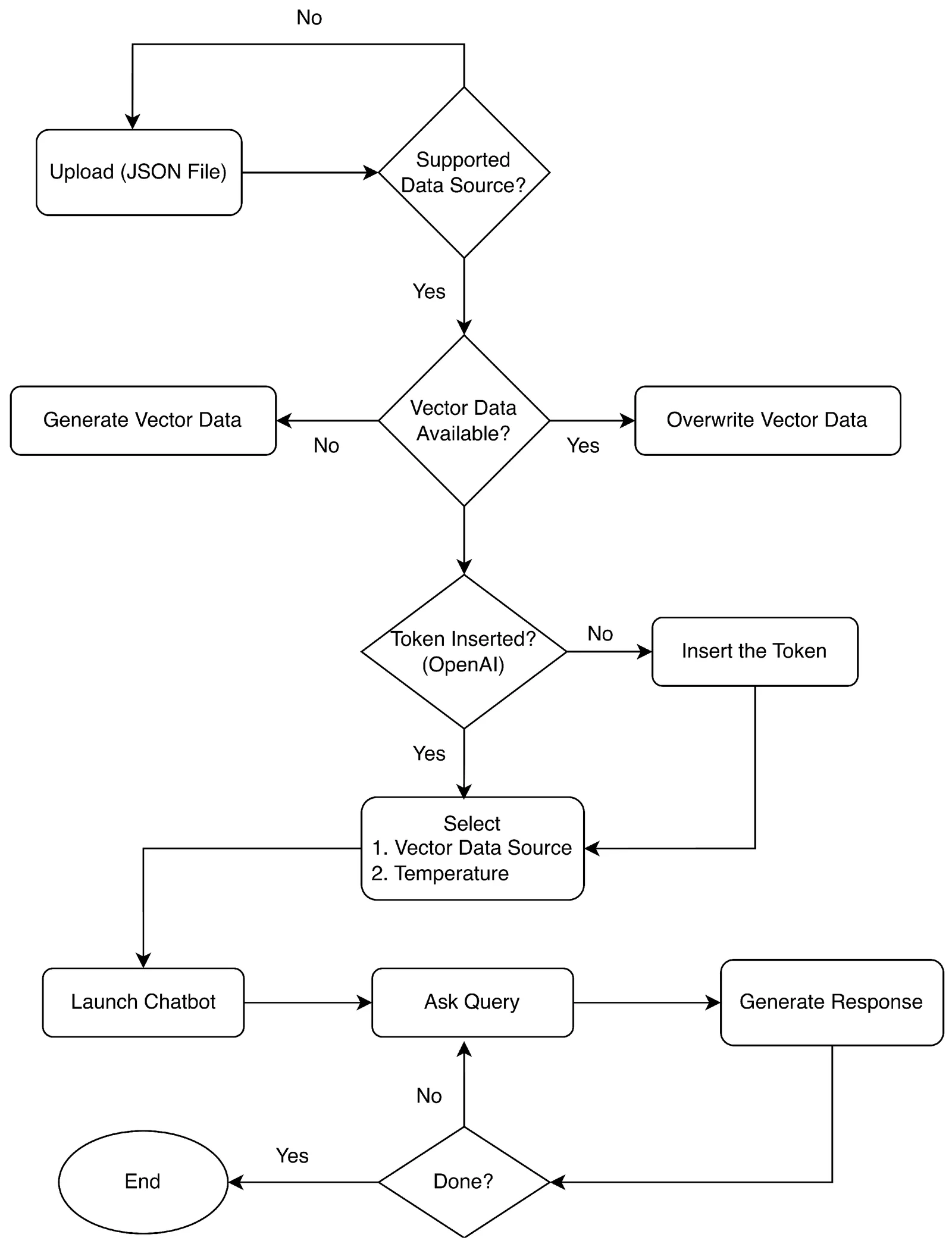
Flowchart of the Retrieval-Augmented Generation (RAG).

Figure 2 represents our RAG system workflow. Initially, we started from the data integration, where it first checks whether the input originates from the supported data source. For our system, we have used JSON format as a supported data source, which includes the anomaly logs with their interpretation, generated by the Stage-2 LLM agents (Section 3.4). Each incident report is one JSON file; only its *interpretation* field is embedded, and the remaining fields are kept as metadata.

The main challenge of the RAG system is: how can vast volumes of external knowledge be managed effectively within the input constraints of LLMs [30]? Traditional methods are like chunking text into smaller, fixed-size segments. But it has some limitations of incomplete retrieval and diminished coherence in generation. We select a unit-based chunking approach for our use case. Specifically, each incident report (one detected event) is treated as an independent chunk, which enables fine-grained retrieval and makes our generated text not biased with one specific unit. For the embedding strategy, we use the OpenAI embedding model and store it in a Faiss vector database for efficient similarity search. In our system, if a vector database already exists, it is overwritten with new data, whereas a new vector database is created when none is available.

In a traditional RAG system, a user query is first embedded, and then used to retrieve the top-*k* most similar documents from the vector database. However, selecting a small number of *k* may result in insufficient contextual information, leading to incomplete or inaccurate answers. On the other hand, choosing a large number of *k* can increase retrieval, but it also increases generation latency and may exceed the input token limitations of LLMs, causing relevant information to be left out. To address this trade-off, our system retrieves the *k* most similar vectors initially from the vector database. These entries are then re-ranked, and the top *k* are selected as the final context. The retriever initially retrieves the 10 most similar reports based on embedding similarity and subsequently applies maximal marginal relevance (MMR) to select the 5 reports used as the final context. This configuration is denoted as Dense + MMR throughout Section 5.6.

**Figure 3:**
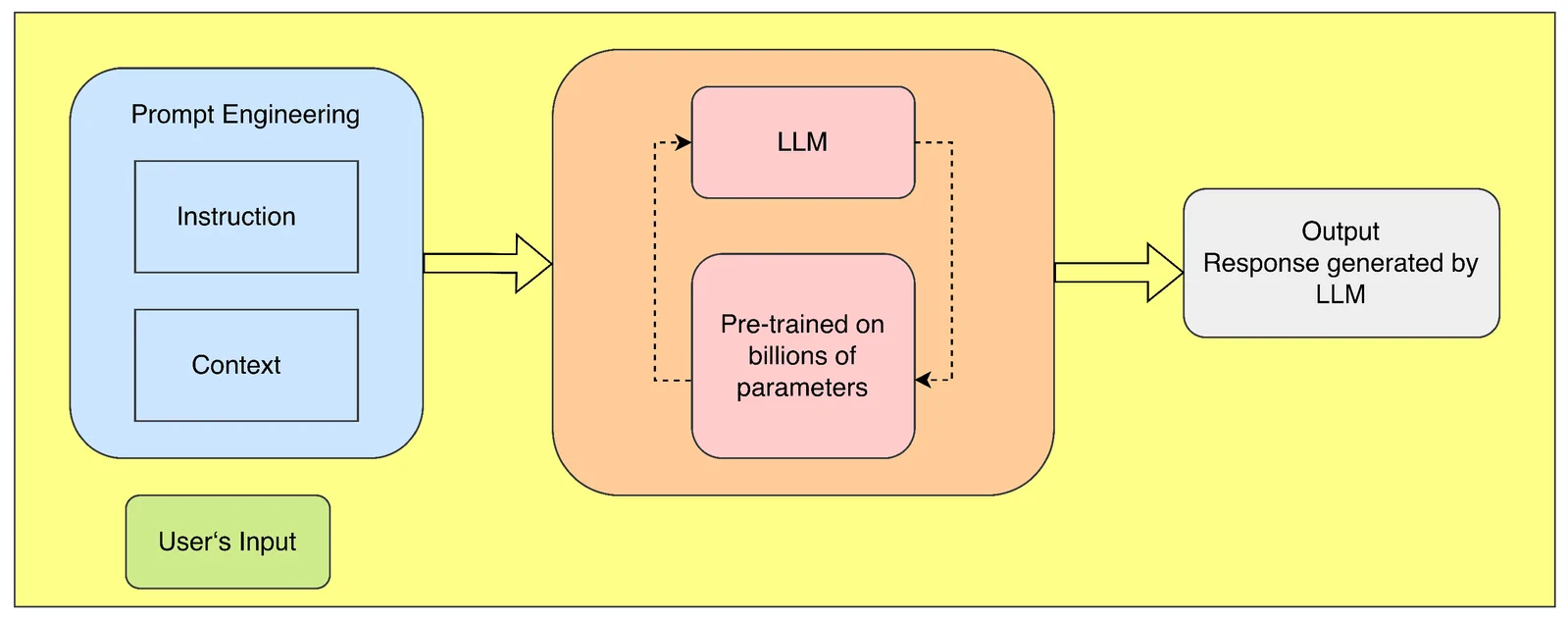
Visual breakdown of prompt engineering components: LLMs trained on extensive data, instruction, and context as pivotal elements shaping the prompt, and a user input interface.

Finally, to generate a response based on the user query and retrieved context, we utilize the pre-trained OpenAI model. To enhance the capability of the pre-trained model, we used prompt engineering, as illustrated in Figure 3. LLMs often stumble in the face of complex reasoning, limiting their potential. Aiming to bridge this gap, in our system, we have used different prompt engineering. It improves the model’s ability to think and explain the answer in a clear and logical order.

***Security considerations.*** The retrieval stage introduces two principal attack surfaces: *prompt injection* and *vector-database poisoning*. Prompt injection refers to malicious content embedded in a retrieved document that may be interpreted by the language model as an instruction rather than as evidence. In RAID, the generator does not receive free-form text from the network; its inputs are limited to the operator query and Stage-2 incident reports generated by the proposed agents from numerical detector outputs under a predefined JSON schema. Retrieved reports are provided within a delimited context block, while the system prompt explicitly instructs the model to treat the retrieved content as evidence only. Generation is performed at temperature 0, and the source reports are returned with each response to support analyst verification. Consequently, an attacker controlling plant telemetry may influence the detector output and, in turn, the reported incident content, but cannot directly inject arbitrary instructions into the generation prompt.

A second threat is *vector-database poisoning*, whereby documents in the FAISS index are inserted or modified to cause misleading evidence to be retrieved for subsequent queries. In the current design, the index is rebuilt in batch from the report directory, and its only external write path is the operator feedback of Figure 1. Therefore, any injected entry would need to conform to the pipeline’s report schema. For operational deployment, write access to the report directory should be restricted to the pipeline service account, and content hashes should be stored with report metadata and verified during index construction. Returning the source reports with each generated response further provides traceability for analyst verification. These measures are considered deployment requirements rather than evaluated security defences; systematic adversarial evaluation of the retrieval stage remains future work.

## 4. Experimental Setting

### 4.1. Anomaly Detection Benchmark Setting

In order to motivate the selection of IF algorithm for further developments, we present a controlled benchmark of nine different unsupervised anomaly detection techniques. The suite of unsupervised algorithms have been evaluated on the HAI 21.03 industrial control system (ICS) dataset and includes: Classic Isolation Forest [31], Extended Isolation Forest (EIF) [32], One-Class SVM [33], Local Outlier Factor [34], Elliptic Envelope (with mandatory PCA pre-reduction), PCA reconstruction error, *k*-NN distance, Autoencoder, and Mahalanobis distance. All algorithms are applied to the same input representation and time alignment. Four recent deep detectors, USAD [35], GDN [36], TranAD [14] and Anomaly Transformer [15], are trained on the raw channels and evaluated with the same calibration and post-processing, without point-adjust, over three seeds.

The raw sensor and actuator values are converted to a common feature space that captures the dynamics of the process, such as control loop and actuator dynamics, thermodynamics, cross-process interactions, states, residual, momentum, and jitter (according to Section 3.1).

To ensure consistency in the output of the final binary values and enable event-based evaluation, a uniform preprocessing framework is used, according to Section 3.2. Specifically, (i) exponentially weighted moving average (EWMA) smoothing is used in all cases during the calibration and testing phase; (ii) a hysteresis thresholding method is used, where the high threshold is used to enter the alarm state and the low threshold is used to leave the state; (iii) short inter-alarm gaps are used to create complete events; and (iv) short event length pruning is used.

Calibration of thresholds is used only on the training data of the normal class (Section 3.2), without attack labels.

***Evaluation metrics.*** Evaluation metrics are based on complementary metrics, including discrimination at the sample, event, and time-aware levels.

*Event-level evaluation.* Because ICS anomalies occur as contiguous intervals, we evaluate detection quality at the *event* level in addition to the sample level. Let $G={\left\{G_{i}\right\}}_{i=1}^{\left|G\right|}$ be the set of ground-truth anomaly segments and $P={\left\{P_{j}\right\}}_{j=1}^{\left|P\right|}$ the set of predicted anomaly segments, where $G_{i}=\left[g_{i}^{s},g_{i}^{e}\right]$ and $P_{j}=\left[p_{j}^{s},p_{j}^{e}\right]$ denote segment start/end indices. Using the overlap indicator I(A∩B≠∅), event recall counts a ground-truth event as detected if *any* predicted segment overlaps it, while event precision counts a predicted event as correct if it overlaps *any* ground-truth event:

(14) $$\begin{array}{cc} R_{\mathrm{evt}} & =\frac{1}{\left|G\right|}\sum_{i=1}^{\left|G\right|}I\;\left(\exists j:\;G_{i}\cap P_{j}\neq \emptyset \right), \end{array}$$

(15) $$\begin{array}{cc} P_{\mathrm{evt}} & =\frac{1}{\left|P\right|}\sum_{j=1}^{\left|P\right|}I\;\left(\exists i:\;P_{j}\cap G_{i}\neq \emptyset \right), \end{array}$$

(16) $$\begin{array}{cc} F_{1}^{\mathrm{evt}} & =\frac{2P_{\mathrm{evt}}R_{\mathrm{evt}}}{P_{\mathrm{evt}}+R_{\mathrm{evt}}}. \end{array}$$

To quantify timeliness, we compute detection delay for each detected ground-truth event as the time between the event start and the first predicted alarm that overlaps it:

(17) $$\begin{array}{cc} \Delta_{i} & =\max\;\left(0,\;p_{j^{🟉}\left(i\right)}^{s}-g_{i}^{s}\right), \end{array}$$

(18) $$\begin{array}{c} \;j^{🟉}\left(i\right)=\min\left\{j:\;G_{i}\cap P_{j}\neq \emptyset \right\}, \end{array}$$

and report the mean delay $\bar{\Delta }$ over detected events.

*Time-aware evaluation.* Finally, we report eTaPR [37] to capture detection quality under labeling ambiguity and partial overlaps typical in real ICS annotations. Let len(A) denote segment length and ov(A,B)=len(A∩B) the number of overlapping samples. For a predicted segment $P_{j}$, define its total overlap with ground-truth events as $\mathrm{OV}\left(P_{j}\right)=\sum_{i}\mathrm{ov}\left(P_{j},G_{i}\right)$ and the overlap ratio $\rho_{j}=\mathrm{OV}\left(P_{j}\right)/\mathrm{len}\left(P_{j}\right)$.

Using a precision-side threshold $\theta_{p}$, eTa-Precision aggregates overlap on the subset of “worthwhile” predicted segments:

(19) $$\begin{array}{c} \eta_{P}=\frac{\sum_{j:\rho_{j}\geq \theta_{p}}\mathrm{OV}\left(P_{j}\right)}{\sum_{j:\rho_{j}\geq \theta_{p}}\mathrm{len}\left(P_{j}\right)}. \end{array}$$

Similarly, for each ground-truth segment $G_{i}$, define its total coverage by predictions as $\mathrm{CV}\left(G_{i}\right)=\sum_{j}\mathrm{ov}\left(G_{i},P_{j}\right)$ and coverage ratio $\kappa_{i}=\mathrm{CV}\left(G_{i}\right)/\mathrm{len}\left(G_{i}\right)$. Using a recall-side threshold $\theta_{r}$, eTa-Recall is:

(20) $$\begin{array}{c} \eta_{R}=\frac{\sum_{i:\kappa_{i}\geq \theta_{r}}\mathrm{CV}\left(G_{i}\right)}{\sum_{i:\kappa_{i}\geq \theta_{r}}\mathrm{len}\left(G_{i}\right)}. \end{array}$$

The corresponding eTa-$F_{1}$ is computed as:

(21) $$\begin{array}{c} \eta_{F1}=\frac{2\eta_{P}\eta_{R}}{\eta_{P}+\eta_{R}}, \end{array}$$

with $\eta_{F1}=0$ when $\eta_{P}+\eta_{R}=0$. As defined above, $\eta_{P}$ and $\eta_{R}$ are computed only over worthwhile segments and detected attacks, so they measure the overlap quality of the detections. Missed attacks and false alarms are captured by the event recall $R_{\mathrm{evt}}$, the event precision $P_{\mathrm{evt}}$ and the FPR, which are always reported with ηF1.

### 4.2. Formal Rules to Natural Language Translation Settings

The language agents were implemented using the GPT-4o model developed by OpenAI, chosen for its proven ability to handle complex structured inputs and produce numerically rigorous explanations [38,39]. As a result of the peculiarities of the architecture adopted, each agent specializes in a distinct explanatory dimension of the anomaly. The prompt engineering strategy adopted to guide these agents reflects the need to overcome the intrinsic limitations of Large Language Models relating to the dilution of technical information and the tendency towards narrative abstraction. First, an explicit quantitative constraint is imposed that forces the model to systematically re-express each mathematical parameter of the decision; this includes directly citing intervention thresholds, directions of inequalities, and the calculation of the numerical margin between the observed value and the decision limit. Second, a rigorous semantic anchorage to the industrial system’s metadata is imposed, integrating variable names, operational descriptions, and units of measurement into the prompts to transform symbolic fluctuations into physically connoted events, such as pressure deviations or anomalies in actuator dynamics. Finally, to eliminate any ambiguity in interpretation, linguistic vagueness is strictly prohibited: the use of qualitative terms is permitted only if supported by specific numerical values, such as standard deviation levels (σ) or probabilities calculated under Gaussian assumptions.

### 4.3. RAG Setting

Table 3 shows the implementation details and hyperparameters of the proposed RAG-based anomaly explanation system. The system architecture is based on a large language model with a vector retrieval function using the FAISS library. It provides a foundation for explaining anomalies based on existing records. The table describes the main architectural components, retrieval parameters, memory settings, and prompts used in our system.

To isolate the contribution of retrieval and enable comparison with simpler retrieval strategies, Section 5.6 evaluates all configurations on a knowledge base comprising 79 incident reports generated by Stage 2 from anomaly events detected by the drift-aware Isolation Forest. We construct a test set of 10 questions, with one question generated using gpt-4o-mini, together with a corresponding reference answer. Generation quality is assessed using faithfulness, answer relevancy, and answer correctness, measured as statement-level F1 against the reference answer. The generation metrics follow the RAGAS definitions [40], with gpt-4o-mini used as the evaluation judge.

## 5. Results

### 5.1. Benchmark Results and Model Selection

The regime-aware analysis recovers two main functional settings (K=2, silhouette value ≈0.306), which is consistent with assumptions about the dataset used (Section 2), despite the low silhouette score, which reflects regimes that overlap because the set points change cyclically. The regimes only split the normal data among local models, with a global fallback model.

**Figure 4:**
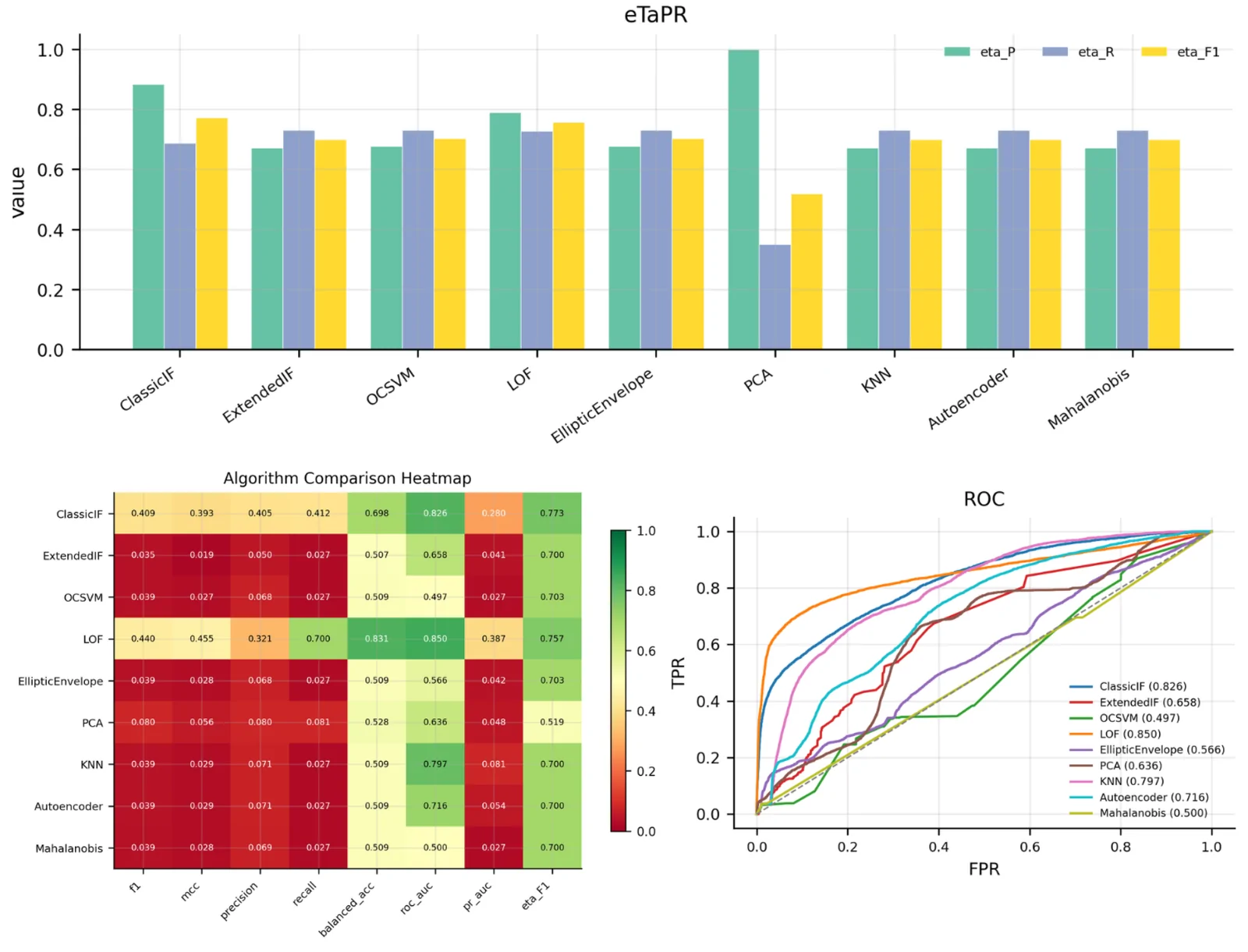
**Benchmark summary on HAI-21.03.** Left: eTaPR metrics (ηP, ηR, ηF1) across detectors. Right: heatmap of normalized point-wise metrics (F1, MCC, precision, recall, balanced accuracy, ROC-AUC, PR-AUC) and ηF1. ClassicIF is the most consistent method across criteria; LOF trades higher recall for increased false alarms and event fragmentation.

Given the benchmark results, ClassicIF exhibits the most balanced and stable performance across the evaluation levels. It achieves a high F1 score of 0.409 and an MCC score of 0.393, with a strong ranking quality (ROC-AUC: 0.826) and the highest temporal accuracy among the competitors (ηF1: 0.773, ηP: 0.884, ηR: 0.687; refer to Figure 4). At the event level, ClassicIF reports the second-highest recall after LOF with 52% (evtR: 0.52), with a delay of approx. 53 samples on average, while still preserving minimal computational costs.

LOF reports the highest F1 score among the competitors (0.440) and the highest event recall (0.96), albeit at the expense of a much more aggressive operating point with a higher FPR (0.038) and a much increased event fragmentation (192), which leads to a much lower evtP (0.214) and ηP (0.789), lower the time-aware score ηF1 (0.757; refer to Figure 4).

The other competitors generally report low recall with corresponding low F1 scores on the point-wise level, despite the relatively high ηF1 scores resulting from the conservative segment outputs. For example, the PCA detector reports a perfect temporal precision (ηP: 1.0) upon firing but with low coverage (ηR: 0.350).

Apart from the accuracy, the ICS deployment requires explanations for the decisions taken by the ICS. Consequently, the ClassicIF detector is chosen as the primary detector with the best temporal performance, while still controlling the false alarm rates and enabling a stable end-to-end interpretability through the predefined rules presented above.

### 5.2. Event-Level Analysis and Comparison with Recent Deep Detectors

**Figure 5:**
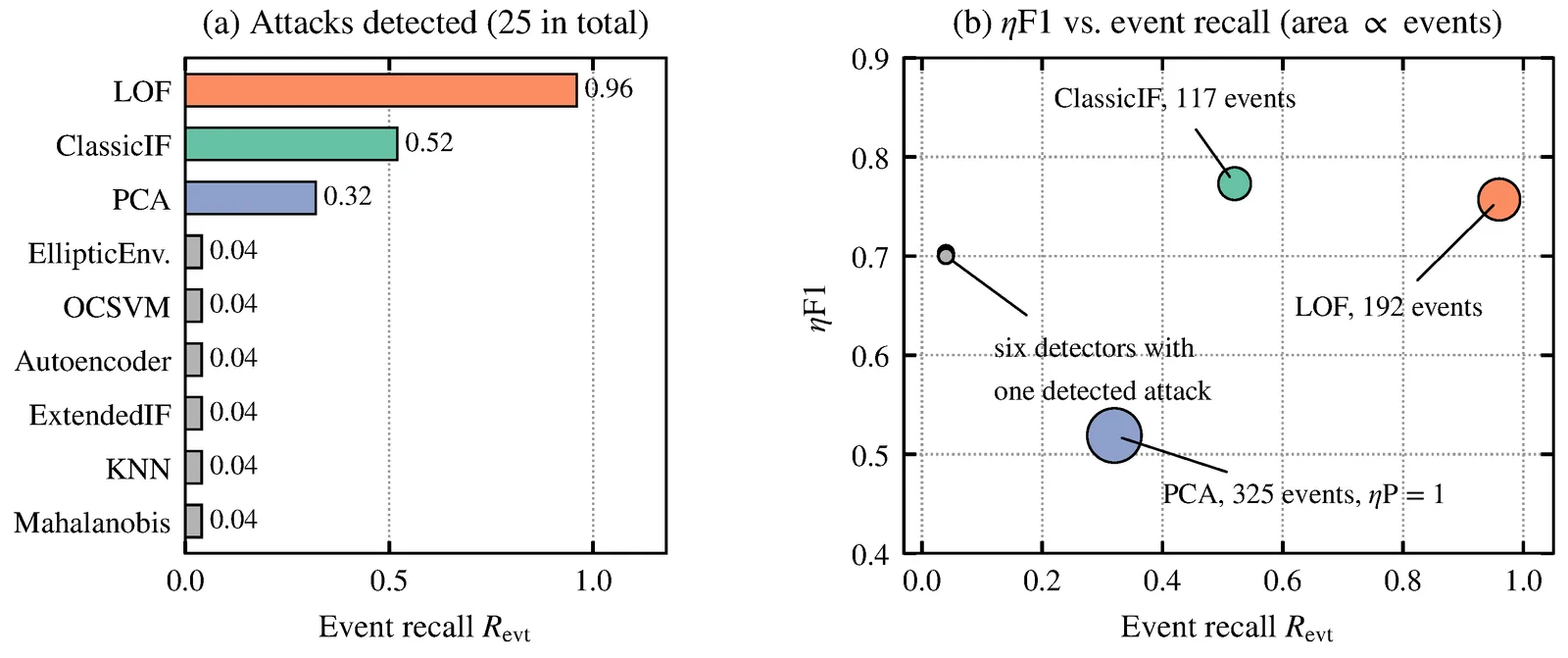
Event-level view of the benchmark (HAI-21.03, 25 attacks). (**a**) Event recall. (**b**) ηF1 against event recall, with marker area proportional to the number of predicted events. Values in Table A1.

Figure 5 shows that only ClassicIF and LOF detect more than one third of the 25 attacks. LOF detects 24 attacks but raises 192 events at a 3.8% FPR, against 117 events and 1.56% for ClassicIF. The six detectors that find a single attack still reach ηF1 ≈0.70, which is why ηF1 is read together with $R_{\mathrm{evt}}$.

**Figure 6:**
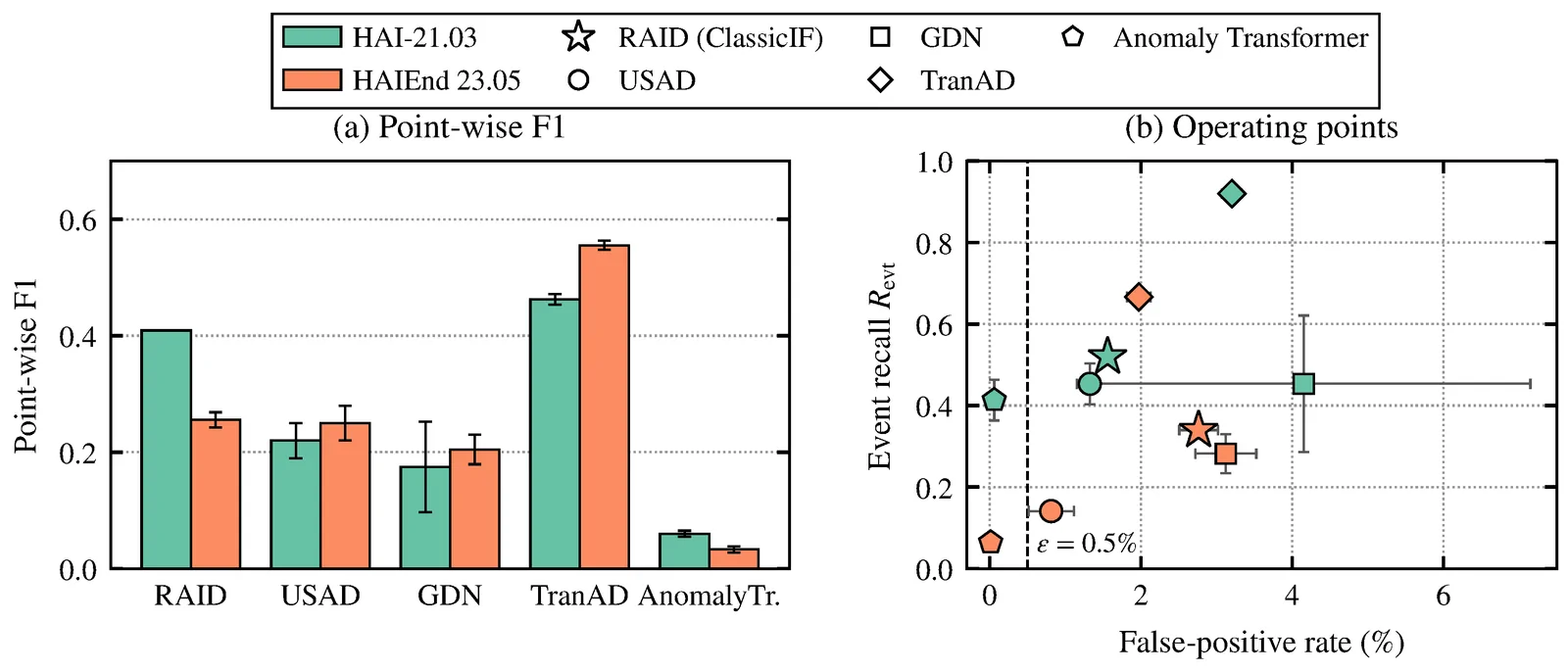
Comparison with recent deep detectors under the same protocol. (**a**) Point-wise F1. (**b**) Event recall against FPR, with the dashed line at the 0.5% target. Deep detectors: mean and standard deviation over three seeds.

Figure 6 compares ClassicIF with the deep detectors. On HAI-21.03, TranAD reaches a higher F1 (0.462 vs. 0.409) but at twice the FPR (3.20% vs. 1.56%), while USAD, GDN and Anomaly Transformer remain below ClassicIF. On HAIEnd 23.05, ClassicIF drops to F1 = 0.255 against 0.555 for TranAD, so the detection stage relies on plant-specific features, while the LLM and RAG stages accept any scoring function.

Overall, Figure 5 and Figure 6 show a trade-off between accuracy and interpretability. TranAD reaches a higher F1 than ClassicIF on both datasets, but at twice the false-positive rate on HAI-21.03 and with an opaque score that does not reveal which signals triggered the alarm. ClassicIF provides the z-scores, counterfactual rules and temporal chains on which the explanation, LLM and RAG stages are built, so every alarm remains traceable to physical quantities. For this reason, the Isolation Forest is kept as the default detector, while a deep model can replace it when accuracy matters more than explanation.

### 5.3. Drift-Aware Isolation Forest

We also examine whether replacing a static decision threshold (introduced in Section 3.2) with a drift-aware dynamic threshold (outlined in Section 3.3) improves the real-world applicability of IF in non-stationary ICS environments.

**Figure 7:**
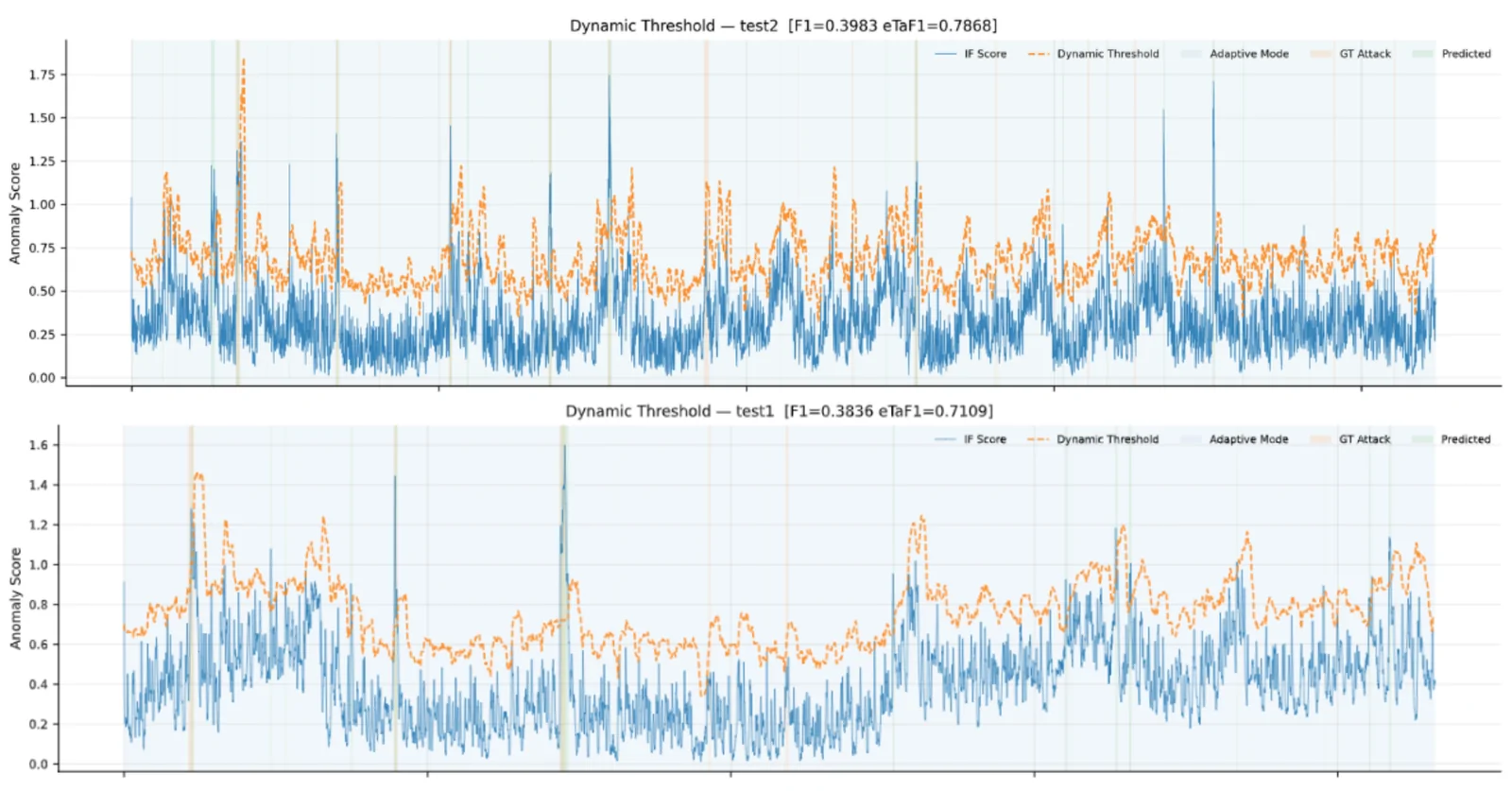
ClassicIF drift-aware (dynamic) thresholding on test2 (**top**) and test1 (**bottom**). The blue curve is the anomaly score; the orange dashed curve is the dynamic threshold. Shaded regions indicate ground-truth attacks and predicted anomaly intervals. The titles report per-file F1 and ηF1.

Figure 7 gives a qualitative overview: the dynamic threshold (orange) reacts to slow changes in the baseline of the anomaly score (blue).

*Benchmark-level comparison.* Compared to the ClassicIF static baseline from the full benchmark, the dynamic configuration shows a performance change of ΔF1=−3.27% and ΔMCC=−3.53%, while the ranking quality is improved by ΔPR-AUC=+13.79% (Table 4).

The time series-aware performance is essentially stable, with a variation of ΔηF1=−0.49%, with ΔηP=−6.56% and ΔηR=+4.81%. In addition, the number of predicted segments decreases, indicating reduced fragmentation, consistent with the more adaptive boundary visible in Figure 7.

*Matched-setting ablation.* To isolate the impact of the thresholding rule itself under an identical score stream, Table 5 reports an internal ablation where the same ClassicIF scoring pipeline is paired with either a static threshold or the dynamic rule. In this controlled setting, the dynamic rule shifts the operating point toward higher coverage, with ΔRecall=+24.68% and ΔηF1=+12.41% (and ΔηR=+20.49%), at the expense of ΔPrecision=−32.51%, ΔF1=−4.78%, ΔMCC=−9.75%, and ΔFPR=+163.83%. Ranking metrics remain unchanged in this matched comparison because thresholding modifies the decision boundary rather than the score ordering.

*Detailed ablation analysis in different test settings.* The performance of the drift-aware thresholding can be seen to vary between the two test sets, as illustrated in the qualitative timelines in Figure 7.

- On test2, the proposed dynamic approach improves time-series-aware performance by ΔηF1=+3.51%, as can be expected given the increased variability in the signal.
- On test1, the performance drops to ΔηF1=−14.87%, as can be expected given the more stable variability in the IF anomaly scores.

The analysis presented here supports the practical applicability of the dynamic method in settings with shifting distributions, within the limits of the HAI testbed. Generally, the adaptive operating point allows for the recovery of anomalous time mass (as identified by high gains in ηF1 under matching conditions), reduces fragmentation in benchmark comparisons, and optimises IDS recall metrics.

In the matched setting, the dynamic rule raises recall by 24.7% but lowers precision by 32.5% and MCC by 9.8%, and raises the FPR from 0.64% to 1.69%. It helps when the score baseline drifts (test2, ηF1 +3.5%) and hurts when it is stable (test1, −14.9%). It is therefore preferable when missed attacks cost more than false alarms, while the static threshold suits stable processes with a strict false-alarm budget.

However, it should be noted that the choice of thresholding technique does not restrict the interpretation of results because of the reliance on independent modules for explanation based solely on the event features.

Therefore, we consider the drift-aware setting to generate LLM-based anomaly interpretations and the retrieval-augmented forensic system, as outlined in the next sections.

### 5.4. Evaluation of Natural Language Rules Extraction Process

The evaluation covers 26 anomalous samples flagged by ClassicIF (12 within attacks and 14 false alarms). For each anomaly provided by the test sets, the system generated an explanation in natural language, whose propositions were evaluated one by one by the Judge Agent. Of these propositions, 1040 in total (26 rules of 40 atomic conditions each, the full decision path of each sample being used as ground truth), 87% were judged to correspond exactly to the mathematical formalism detected as the starting reference (type 1). 9%, on the other hand, were labeled as misinterpretations (type 2). The remaining 4% were labeled as not associated with any rule (type 3). The introduction of rationales within the structured output allows errors to be analyzed in an attempt to highlight recurring error patterns and prepare policies to mitigate them. This type of analysis shows that one of the main reasons why a correspondence is judged to be type 2 is the loss of numerical precision. This is due to semantic over-interpretation during the transformation from mathematical formalism to natural language. This category includes unjustified rounding and improper conversions to percentages. Generalizations of specific phenomena, such as causal attributions not explicitly supported by ground truth rules, are also labeled as type 2 errors. It is worth noting, however, that this particular type of phenomenon could derive from the additional information provided to agents regarding the meaning and operational range of each variable and the associated physical phenomenon. As for type 3 errors, they can be traced back to two macro-dynamics: (i) A tendency toward interpretative generalization that expands the numerical content toward causal or systemic inferences that are not formally justified; in these cases, the model semantically expands the available information, transforming co-occurrences or local numerical values into systemic or causal narratives. It is not a mistake in understanding the data, but an inferential expansion beyond the formally justified content. (ii) A structural misalignment between generated propositions and precise mathematical rules, which prevents a deterministic association between statement and ground truth. In this case, the problem is not so much abstraction, but the lack of explicit correspondence between proposition and formal rule, which prevents the Judge from establishing a deterministic alignment. Overall, one of the causes of the problems identified in this paragraph is undoubtedly the natural tendency of linguistic models to favor narrative coherence and causal plausibility over quantitative fidelity; frequently, errors do not stem from a lack of local understanding of the data, but from a distortion introduced in the transition from rigorous symbolic representation to the construction of a coherent interpretative discourse.

### 5.5. Evaluation of Explanation Quality

Evaluating the performance of a Retrieval-Augmented Generation (RAG) system is challenging due to the complexity of grounding generated explanations in retrieved context and ensuring both factual accuracy and relevance [40]. In order to overcome this challenge, the Retrieval Augmented Generation Assessment (RAGAS) has been used to evaluate the various dimensions of the RAG system without relying on human annotations. The performance of the RAG system was evaluated by using the faithfulness and answer relevancy metrics. Faithfulness measures how well the answer has been grounded by the context, whereas answer relevancy measures how well the answer has addressed the query.

*Prompt Engineering Evaluation.* To comprehensively assess our system, we employ multiple prompt engineering strategies, including Chain-of-Thought (CoT) prompting [41], Self-Consistency [42], Least-to-Most Prompting [43], ReAct (Reason + Act) [44], and Tree-of-Thought (ToT) reasoning [45]. The aggregated RAGAS evaluation scores (averaged over five questions) for five prompting strategies are presented in Table 6, using *Faithfulness* and *Answer Relevancy* as evaluation metrics. The highest Faithfulness score was achieved by Self-Consistency (95.7), with Chain-of-Thought ranking second with 92.8. Conversely, ReAct (Reason+Act) had the highest Answer Relevancy (74.2), reflecting greater answer alignment with user questions. The performance of Least-to-Most Prompting was found to be lower than that of other strategies on both metrics: 81.8 for Faithfulness and 53.2 for Answer Relevancy.

Tree-of-Thought had the lowest Faithfulness (75.3) with moderate Answer Relevancy (64.1). The results imply a trade-off between Faithfulness and Answer Relevancy. The prompting comparison above is restricted to five questions and to the proposed retriever; the next subsection extends the evaluation to all 10 questions and adds retrieval baselines.

### 5.6. Evaluation of Retrieval Strategies and Baselines

To assess the role of retrieval and compare the proposed approach with simpler retrieval strategies, we evaluate five configurations: (i) the LLM without retrieved context; (ii) Okapi BM25 retrieval with the top 5 documents; (iii) dense similarity retrieval with the top 1 document; (iv) dense similarity retrieval with the top 5 documents; (v) the proposed Dense + MMR configuration. The resulting generation quality is reported in Table 7.

The LLM-only configuration, which does not receive any retrieved context, yields very low answer relevancy and correctness scores of 0.016 and 0.004, respectively. The proposed Dense + MMR configuration achieves a faithfulness score of 0.998, an answer relevancy score of 0.774, and an answer correctness score of 0.782. These results demonstrate the importance of providing incident-specific contextual information to the generator when answering questions grounded in the plant-specific knowledge base.

The comparison between dense top 1 and dense top 5 retrieval further indicates that increasing the amount of retrieved context can improve generation quality. Increasing the context from one to five reports raises answer relevancy from 0.262 to 0.433 and answer correctness from 0.276 to 0.420. With MMR-based re-ranking, answer correctness reaches 0.782. Overall, the results indicate that both the quality of retrieved documents and the selection of the final contextual reports influence the performance of the RAG pipeline.

***Qualitative panel.*** Table 8 reports the answer-correctness scores for the ten evaluated questions across the proposed Dense + MMR pipeline, BM25, and the LLM without retrieval. The proposed pipeline achieves correctness scores ranging from 0.74 to 0.82 across the ten questions, with an average of 0.782. In comparison, BM25 obtains scores between 0.30 and 0.50, with an average of 0.433, while the LLM-only configuration obtains a mean correctness of 0.004. These results indicate that providing retrieved incident reports substantially improves the ability of the generator to produce answers grounded in the plant-specific knowledge base. The proposed Dense + MMR configuration consistently achieves higher correctness than BM25 across all ten questions, illustrating the benefit of combining dense semantic retrieval with MMR-based context selection.

## 6. Conclusions

This article proposes RAID, a tripartite framework combining the best of retrieval-augmented intrusion detection, with applications in cyber-physical systems and industrial control systems. It offers powerful unsupervised intrusion detection, traceable explanations, and a queryable forensic memory. The first stage of the framework combines physics-informed feature engineering with regime identification, along with robust unsupervised Isolation Forest, EWMA smoothing, and clean window adaptive thresholding. The second stage offers structured data converted into natural language incident reports. The third stage constructs a RAG store, enabling operator queries based on previous incidents.

When the algorithm is applied to the HAI-21.03 benchmark, the best overall detection performance in terms of stability is achieved with F1=0.409, MCC=0.393, and ROC-AUC=0.826. The best performance in terms of time series detection using the eTaPR algorithm is achieved with ηF1=0.773, ηP=0.884, and ηR=0.687. The dynamic thresholding process improves performance in terms of time series recall (ηR), thereby demonstrating the effectiveness of the proposed framework in improving the accuracy of time series detection by dynamically adapting thresholds to the drift. The explanation layer shows high fidelity, with 87% of the 1,040 propositions precisely correct according to the underlying formalism, and 9% misinterpreted. Finally, different prompt engineering settings for the RAG-based system are evaluated to assess faithfulness and answer relevance; this benchmark is dominated by the Self-Consistency setting. Across ten evaluated questions, the proposed Dense + MMR pipeline improves mean answer correctness to 0.782, compared with 0.433 for BM25 and 0.004 for the LLM without retrieval, while achieving a faithfulness score of 0.998. These results demonstrate the benefit of retrieval and MMR-based context selection for generating answers grounded in knowledge base.

Compared with recent deep detectors, ClassicIF trades some F1 against TranAD for a lower false-positive rate and native explanations, and on HAIEnd 23.05 its accuracy drops without plant-specific features.

Future work could include plugging sequence-based detectors into the explanation stage, human-in-the-loop constraints, and causal relationships to further enhance it. On the retrieval side, future work will investigate a hybrid BM25–dense retriever, a hand-verified question set formulated without record identifiers, and an adversarial evaluation of the retrieval stage against prompt-injection and vector-database-poisoning attacks.

## Figures and Tables

**Table 1:** Physics-informed feature groups.

Grp	Name	Dim	Detects
1	Control-Loop	20	Sensor injection, setpoint alteration
2	Actuator	25	Command injection
3	Thermodynamic	6	Cross-sensor inconsistency
4	Cross-Process	4	Inter-process coupling anomalies
5	State/Discrete	∼12	Saturation, regime shifts
6	Generic Residuals	∼260	Time-aware spectrum
	**Total**	∼**330**	

**Table 2:** Structured explanation components.

#	Component	Question Answered
1	Z-Score Analysis	Which features are most abnormal?
2	Counterfactual	Which features *impact* the anomaly score?
3	CF Rules	What condition defines this anomaly?
4	Contrastive	How does it differ from normal?
5	Temporal Chain	How did the anomaly propagate?
6	Redundancy	Which features overlap?

**Table 3:** RAG Configuration for Anomaly Explanation System.

Component	Configuration
JSON Processing	Extract “Interpretation” field only
LLM	gpt-4o-mini
Embedding Model	text-embedding-3-small
Vector Store	FAISS
Chunking Method	One incident report as one chunk
Temperature	0.0
Top-*k* Retrieval	5
Fetch-*k*	10
Chain Type	stuff
Return Source Docs	Enabled
Memory Window (*k*)	0
Prompting Strategy	RAG-based reasoning prompt
Knowledge base	79 incident reports (one per detected event)

**Table 4:** ClassicIF full-benchmark static baseline vs. drift-aware (dynamic) thresholding. Δ%=100·(Dynamic−Static)/Static.

Metric	Static	Dynamic	Δ (%)
F1	0.4086	0.3952	−3.27%
MCC	0.3932	0.3793	−3.53%
Precision	0.4053	0.3837	−5.34%
Recall	0.4120	0.4076	−1.07%
FPR	0.0156	0.0169	+8.32%
PR-AUC	0.2801	0.3188	+13.79%
ηP	0.8837	0.8257	−6.56%
ηR	0.6872	0.7203	+4.81%
ηF1	0.7732	0.7694	−0.49%
#Pred seg.	117	79	−32.48%

**Table 5:** Matched-setting ablation: static vs. drift-aware (dynamic) thresholding under the same ClassicIF scoring pipeline. Δ%=100·(Dynamic−Static)/Static.

Metric	Static	Dynamic	Δ (%)
F1	0.4151	0.3952	−4.78%
MCC	0.4203	0.3793	−9.75%
Precision	0.5684	0.3837	−32.51%
Recall	0.3269	0.4076	+24.68%
FPR	0.0064	0.0169	+163.83%
ηP	0.8006	0.8257	+3.14%
ηR	0.5978	0.7203	+20.49%
ηF1	0.6845	0.7694	+12.41%
#Pred seg.	27	79	+192.59%

**Table 6:** Aggregated RAGAS evaluation results over five questions.

Prompting Strategy	*Faithfulness*	*Answer Relevancy*
Chain-of-Thought (CoT)	92.8	54.3
Self-Consistency	**95.7**	64.5
Least-to-Most Prompting	81.8	53.2
ReAct (Reason + Act)	80.7	**74.2**
Tree-of-Thought (ToT)	75.3	64.1

**Table 7:** Generation quality over all 10 questions. All retrieval configurations use the same zero-shot grounded prompt. The Best Score for Each Metric Is Marked in **Bold**.

Configuration	Faith.	Relev.	Correct.
LLM only (no retrieval)	–	0.016	0.004
BM25 top-5	0.912	0.422	0.433
Dense top-1	0.938	0.262	0.276
Dense top-5	0.899	0.433	0.420
Dense + MMR (proposed)	**0.998**	**0.774**	**0.782**

**Table 8:** Answer correctness for the ten evaluated questions across the considered configurations. The Best Score for Each Metric Is Marked in **Bold**.

#	Question (Abridged)	Proposed	BM25	LLM Only
1	z-scores of PCV02 and turbine manual speed demand	**0.75**	0.40	0.00
2	z-scores of P4_HT_LD and P1_B4002__mom	**0.80**	0.45	0.00
3	scheduled power demand of hydro and steam turbine models	**0.78**	0.35	0.00
4	FCV02 command rate and water-outflow setpoint momentum	**0.82**	0.50	0.00
5	P1_B4002__mom and P1_B4002__aj	**0.76**	0.40	0.00
6	pressure acceleration and water-level tracking error	**0.79**	0.45	0.00
7	P1_B400B__aj and actuator FCV01	**0.74**	0.30	0.00
8	water-outflow setpoint versus actual outflow	**0.81**	0.48	0.00
9	z-scores of P4_HT_LD and P3_LCV01D	**0.77**	0.50	0.00
10	z-scores of xp_coh_level and scheduled power demands	**0.80**	0.50	0.04

## Data Availability

The data utilized in this study are publicly available and can be accessed at https://github.com/icsdataset/hai (access on 20 October 2026).

## Appendix A. Detailed Detection Metrics

**Table A1:** Event-level metrics of the benchmark (HAI-21.03, 25 attacks): FPR, event precision and recall, number of predicted events, mean detection delay (s) and time-aware scores.

Detector	FPR (%)	$P_{\mathrm{evt}}$	$R_{\mathrm{evt}}$	#Events	Delay	ηP	ηR	ηF1
ClassicIF	1.56	0.188	0.52	117	53.3	0.884	0.687	0.773
ExtendedIF	1.32	0.040	0.04	25	0.0	0.673	0.730	0.700
OCSVM	0.97	0.042	0.04	24	0.0	0.677	0.730	0.703
LOF	3.82	0.214	0.96	192	20.3	0.789	0.727	0.757
EllipticEnvelope	0.96	0.037	0.04	27	0.0	0.677	0.730	0.703
PCA	2.42	0.031	0.32	325	61.5	1.000	0.350	0.519
KNN	0.92	0.037	0.04	27	0.0	0.673	0.730	0.700
Autoencoder	0.92	0.037	0.04	27	0.0	0.673	0.730	0.700
Mahalanobis	0.95	0.042	0.04	24	0.0	0.673	0.730	0.700

**Table A2:** Deep detectors under the same protocol (0.5% target FPR, no point-adjust). Deep models: mean ± std over three seeds.

Detector	F1	MCC	FPR (%)	ROC-AUC	$P_{\mathrm{evt}}$	$R_{\mathrm{evt}}$	ηF1
*HAI-21.03*							
ClassicIF	0.409	0.393	1.56	0.826	0.188	0.520	0.773
USAD	0.220 ± 0.030	0.207 ± 0.032	1.32	0.805	0.368	0.453	0.578 ± 0.016
GDN	0.175 ± 0.078	0.155 ± 0.080	4.15	0.732	0.299	0.453	0.635 ± 0.016
TranAD	0.462 ± 0.009	0.469 ± 0.008	3.20	0.856	0.288	0.920	0.750 ± 0.002
Anomaly Transf.	0.060 ± 0.005	0.132 ± 0.018	0.06	0.558	0.577	0.413	0.264 ± 0.059
*HAIEnd 23.05*							
ClassicIF	0.255 ± 0.013	0.226 ± 0.015	2.76	0.704	0.207	0.340	0.745 ± 0.005
ExtendedIF	0.352 ± 0.001	0.325 ± 0.001	2.69	0.747	0.230	0.577	0.766 ± 0.001
LOF	0.171	0.163	1.36	0.640	0.262	0.212	0.522
USAD	0.250 ± 0.030	0.268 ± 0.046	0.81	0.613	0.319	0.141	0.754 ± 0.031
GDN	0.205 ± 0.025	0.173 ± 0.028	3.12	0.656	0.118	0.282	0.682 ± 0.009
TranAD	0.555 ± 0.008	0.537 ± 0.008	1.97	0.847	0.332	0.667	0.836 ± 0.001
Anomaly Transf.	0.033 ± 0.005	0.115 ± 0.010	0.01	0.533	0.841	0.064	0.000
